# Crystalline Quality, Composition Homogeneity, Tellurium Precipitates/Inclusions Concentration, Optical Transmission, and Energy Band Gap of Bridgman Grown Single-Crystalline Cd_1−x_Zn_x_Te (0 ≤ x ≤ 0.1)

**DOI:** 10.3390/ma14154207

**Published:** 2021-07-28

**Authors:** Ana María Martínez, Paula Giudici, Alicia Beatriz Trigubó, Raúl D’Elía, Eduardo Heredia, Rodrigo Ramelli, Rubén González, Felipe Aza, Ulises Gilabert

**Affiliations:** 1 Unidad de Investigación y Desarrollo Estratégico para la Defensa (Consejo Nacional de Investigaciones Científicas y Técnicas-Ministerio de Defensa) UNIDEF, Buenos Aires B1603ALO, Argentina; anamartinez.tutora@gmail.com (A.M.M.); rdelia@citedef.gob.ar (R.D.); eheredia@citedef.gob.ar (E.H.); 2 Facultad Regional Buenos Aires-Universidad Tecnológica Nacional FRBA-UTN, Buenos Aires C1179AAQ, Argentina; ulises.gilabert@segemar.gov.ar; 3 Comisión Nacional Energía Atómica-Centro Atómico Constituyentes CNEA CAC, Instituto de Nanociencia y Nanotecnología-INN, San Martin B1650KNA, Argentina; giudici@tandar.cnea.gov.ar; 4 CNEA-CAC, Unidad Actividad Materiales, Laboratorio de Microanálisis, San Martin B1650KNA, Argentina; ramelli@cnea.gov.ar (R.R.); rugonz@cnea.gov.ar (R.G.); 5 Instituto de Tecnología Minera-Servicio Geológico Minero Argentino INTEMIN-SEGEMAR, San Martin B1650WAB, Argentina; felipe.aza@segemar.gov.ar

**Keywords:** Cd_1−x_Zn_x_Te (0 ≤ x ≤ 0.1), Bridgman method, chemical etching, EPMA, DSC, FTIR, PL

## Abstract

Cd_1−x_Zn_x_Te (0 ≤ x ≤ 0.1) ingots were obtained by Bridgman’s method using two different speeds in order to find the optimal conditions for single-crystalline growth. Crystalline quality was studied by chemical etching, the elemental composition by wavelength dispersive spectroscopy (WDS), tellurium (Te) precipitates/inclusions concentration by differential scanning calorimetry (DSC), optical transmission by Fourier transformed infrared spectrometry (FTIR), and band gap energy (Egap) by photoluminescence (PL). It was observed that the ingots grown at a lower speed were those of the best crystalline quality, having at most three grains of different crystallographic orientation. The average dislocations density in all of them were similar and correspond to materials of good quality. EPMA results indicated that the homogeneity in the composition was excellent in the ingots central part. The concentration of Te precipitates/inclusions in all ingots was below the instrument (DSC) detection limit, 0.25% wt/wt. In the case of wafers from Cd_0.96_Zn_0.04_Te and Cd_0.90_Zn_0.10_Te ingots, the optical transmission was better than that of commercial materials and varied between 60% and 70%, while for pure CdTe, the transmission range was between 50% and 55%, the latter being decreased by the presence of Te precipitates/inclusions. The band gap energy Eg of different wafers was experimentally obtained by PL measurements at 76 K. We observed that Eg increased with the Zn concentration of the wafers, following a linear regression comparable to those proposed in the literature, and consistent with the results obtained with other techniques.

## 1. Introduction

Cd_1−x_Zn_x_Te (0 ≤ x ≤ 0.1) (CZT) semiconductors, having an energy gap greater than 1.4 eV, are important in the manufacture of X-ray and gamma detectors (γ) [1,2], and because they can operate at room temperature, they present advantages compared to those of Si or Ge that require operating temperatures between 77 and 150 K. The compounds are also used as a substrate for the growth of epitaxial films sensitive to IR radiation [3,4].

CZT was initially used in solid-state devices such as solar cells, photodetectors, and light-emitting diodes. In recent years, CZT detector array technology has enabled room temperature X-ray and gamma imaging for applications in nuclear medicine, space science, and national defense (control of radioactive materials, weapons, and nuclear plants) [5,6,7].

A good detector needs high crystalline quality and precise band gap values. The high crystallinity is directly related with the compound homogeneity and low amount of dislocations as well as the presence of Te precipitated/inclusions [8]. The homogeneity of the composition is also important to guarantee uniformity in electrical resistivity and energy bandgap [9].

These materials are usually fabricated by the vertical Bridgman (VB), horizontal Bridgman (HB), high-pressure Bridgman technique, vertical gradient freeze (VGF) method, or traveling heater method (THM). Most of them are sophisticated techniques, expensive, or of difficult implementation [10,11,12,13,14]. For this reason, the challenge for developing countries is to find a low-cost fabrication method, like vertical Bridgman, and with acceptable material quality.

At UNIDEF, CZT single-crystalline ingots are grown by Bridgman’s method at different speeds to find the optimal growing conditions. The materials were characterized by chemical etching, wavelength-dispersive spectroscopy (WDS), differential scanning calorimetry (DSC), Fourier transformed infrared spectrometry (FTIR) to analyze the crystalline quality, and photoluminescence (PL) to determine the band gap energy. In particular, we investigated the presence of Te precipitates/inclusions in the CZT, one of the principal causes of detector devices damage.

It was observed that the ingots grown at a lower speed present the best crystalline quality for having at most three grains of different crystallographic orientation. A direct relation between the band gap, CZT composition, and growth velocity was found. We demonstrate that Bridgman’s technique is suitable in achieving high-quality crystalline ingots, with large single-crystalline volumes and high homogeneity. This growth technique can be used to fabricate the above-mentioned detectors [15].

## 2. Experimental

The starting elements (Cd, Zn, and Te) were commercially acquired with a purity of 99.9999%. Subsequently, each one was purified three times by dynamic vacuum distillation [16,17,18] to reduce impurities and oxides in the elements.

The ingots were grown using the Bridgman’s method, which consists of fractional solidification of the previously melted alloy while passing through a temperature gradient of 10 °C/cm [19,20]. Two growth speeds were used: 1.66 and 3.32 mm/h.

The crystallographic [111] orientation of the ingots was determined by the Laue’s technique (Philips PW3710, Amsterdam, The Netherlands). The 2-mm thick wafers were cut by aligning the ingots [111] direction with the wire saw (South Bay Technology, San Clemente, CA, USA). These wafers were mechanically polished (Büehler-Minimet, Lake Bluff, IL, USA) with alumina (Alpha Alumina Powder agglomerate free, Leco Instruments Ltd., Mississauga, Canada) to get soft and planar surfaces. First, 9 μm and afterward 1 μm of alumina powder were used. A bromine-ethylene glycol 1% *v/v* solution [21] was employed in the chemical polishing to eliminate the damage from mechanical polishing.

Crystalline quality was studied by chemical etching. Nakagawa solution (3HF:2H_2_O_2_:2H_2_O) [22,23] was used to reveal dislocations in the [111] Cd crystallographic plane. The determination of the density of dislocations and misorientation between contiguous subgrains was performed by counting defects on optical micrographs (Union Versamet 5279 metallographic optical microscope- San Jose, CA, USA) applying the Read Shockley approximation [24].

The elemental composition was determined using a Cameca SXFive Electron Probe Micro Analyzer (EPMA, Paris, France) The diameter of the beam focused on the surface of the sample was 0.2–0.4 µm. The interaction volume of the electrons of the beam with the material was approximately 1 µm^3^. Standards of Cd, Zn, and Te, extracted from the previously purified material, were used.

The differential scanning calorimetry (DSC, New Castle, DE, USA) technique was used to determine the concentration of Te precipitates/inclusions using simultaneous thermal analysis equipment TA-Q600 (SEGEMAR). The thermographs were made in alumina crucibles and argon atmosphere with a heating speed of 10 °C/min. The calibration of the baseline was carried out with a sapphire standard and those of the temperature and cell constant with Zn (99.99999) [25].

The optical transmission measurements were performed using a Fourier transformed infrared (IR) spectrometer (FTIRS) Perkin Elmer System 2000 model (Richmond, CA, USA), in the spectral range of 2 to 27 microns. Transmittance gives an idea of the amount of defects, since the better the crystallographic quality, the higher the transmittance [26].

The band gap energy (Eg) was estimated from the photoluminescence emission, obtained in backscatter geometry using a Horiba Jobin Yvon Lab RAM HR-800 spectrometer (Kyoto, Japan) with a diffraction grating of 600 lines/nm (Kyoto, Japan), cooling the samples in a liquid N_2_ cryostat. The used excitation source was a He-Ne laser with a line of 632.8 nm.

## 3. Results and Discussion

### 3.1. Crystalline Quality Determination by Chemical Etching

**Figure 1:**
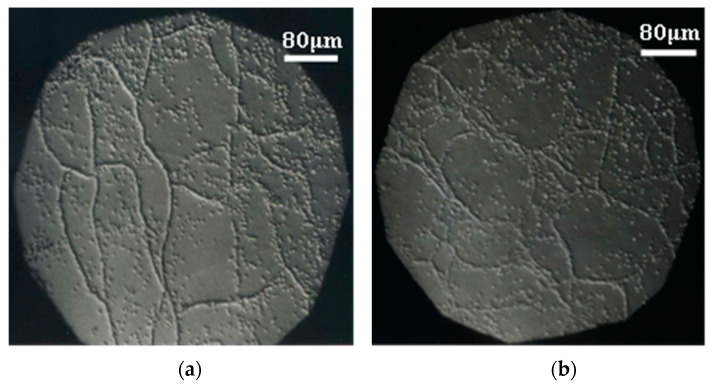
(**a**) CdTe, v = 3.32 mm/h, δ = 1.2 × 10^6^ cm^−2^, ϕ = 23″; (**b**) CdTe, v = 1.66 mm/h, δ = 1.3 × 10^6^ cm^−2^, ϕ = 24″.

**Figure 2:**
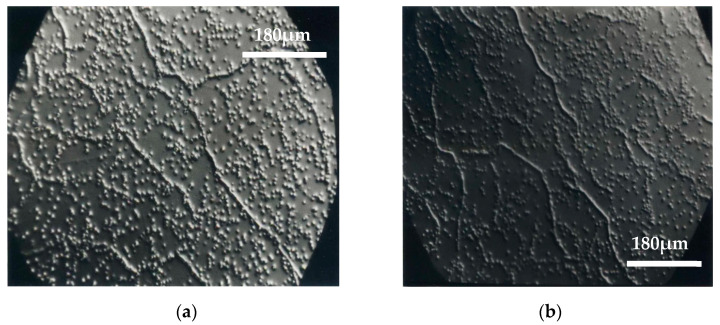
(**a**) Cd_0.96_Zn_0.04_Te, v = 3.32 mm/h, δ = 6.3 × 10^5^ cm^−2^, ϕ = 19″; (**b**) Cd_0.96_Zn_0.04_Te, v = 1.66 mm/h, δ = 7.6 × 10^5^ cm^−2^, ϕ = 25″.

**Figure 3:**
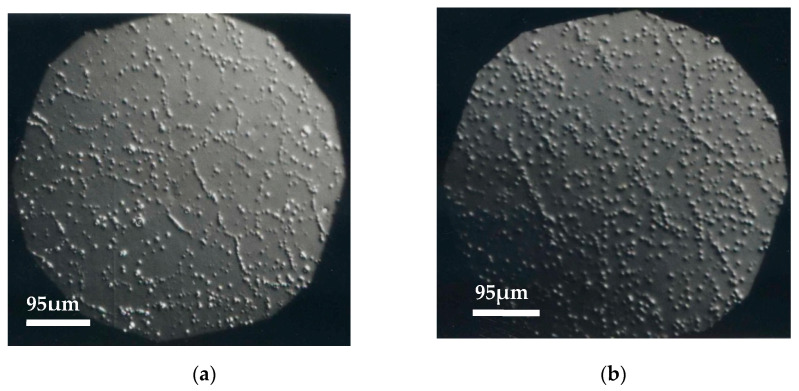
(**a**) Cd_0.90_Zn_0.10_Te, v = 3.32 mm/h, δ = 6.6 × 10^5^ cm^−2^, ϕ = 26″; (**b**) Cd_0.90_Zn_0.10_Te, v = 1.66 mm/h, δ = 6.9 × 10^5^ cm^−2^, ϕ = 19″.

Some of the optical micrographs of the wafers surface obtained from ingots grown at different speeds and different concentrations are shown. The dislocations distribution and the subgrain structure can be observed due to the presence of corrosion pits (Figure 1, Figure 2 and Figure 3). Dislocations density measurement is a good indicator of device feasibility, since dislocations in the material decrease the detector performance [27]. Table 1 shows for each ingot the composition, growth rate, and the number of grains, and also includes the average results of the dislocations density and contiguous subgrains misorientation, obtained from several wafers and ingots.

**Figure 4:**
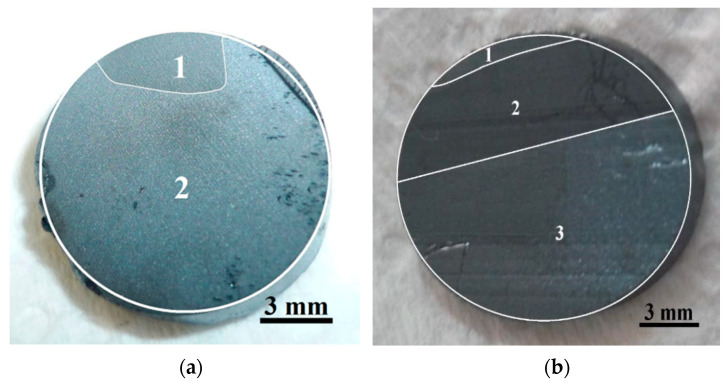
(**a**) Wafer of a Cd_0.90_Zn_0.10_Te ingot grown at 1.66 mm/h (orientation unknown); (**b**) [111] wafer of a Cd_0.96_Zn_0.04_Te ingot grown at 3.22 mm/h.

Dislocations are fundamentally generated by residual stress. Te precipitates/inclusions form during the growth process producing additional volumes in the crystals, which generates large elastic stress, resulting in the formation of dislocations during the subsequent cooling process [28,29]. Our results show that the ingots grown at a lower speed are of the best crystalline quality: CdTe has three grains of different crystallographic orientation whereas only two grains are observed in the ingots of CdTe alloyed with Zn. The ingots grown at a higher speed show 4 or 5 grains, which is also a good result for some applications (Table 1 and Figure 4). These results are comparable to those described by U. Gilabert et al. [30] and T.E. Schlesinger et al. [31].

Nakagawa’s solution was employed on face A (Cd), since it had been proved, comparing with X-rays results, that each corrosion figure corresponded to a dislocation [22,23]. On face B (Te), instead, the etching solution gave lower average values of the dislocation density [32,33]. This was observed in all our wafers and is coincident with reports in the literature, so the information from face B was discarded.

It is generally observed that when increasing the Zn content in the CdTe alloy, the dislocations density and contiguous subgrains misorientation decrease, as result of an increase of CdTe hardness with Zn.

Moreover, in the case of CdTe alloyed with 10 at% Zn, contiguous subgrains misorientation increases with the growth rate. The ingots grown at a lower speed (1.66 mm/h) achieve a better thermal equilibrium and, consequently, present a higher crystalline quality.

### 3.2. Data Analysis of the EPMA Measurements

#### 3.2.1. Analysis of Homogeneity in Composition

**Figure 5:**
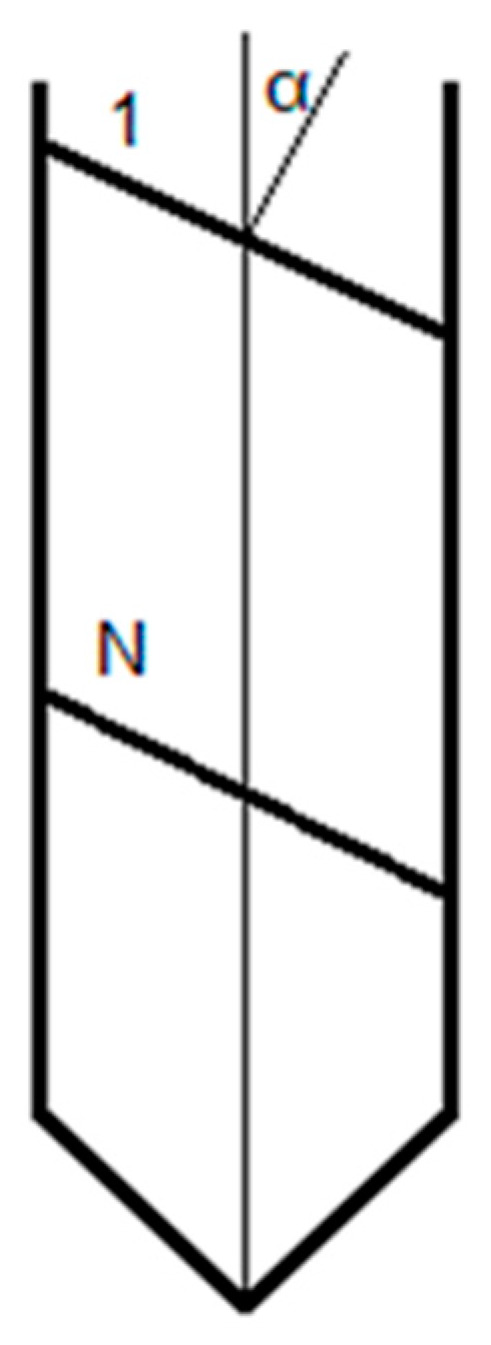
Outline of the ingot in the ampoule with [111] wafers orientation.

The atomic fraction of Zn, Cd, and Te of several Cd_1−x_Zn_x_Te wafers (0 ≤ x ≤ 0.1) had been measured in order to study the effect of segregation. Figure 5 schematizes the ampoule containing the ingot. The first wafer is symbolized as “1”, near the top of the ingot (shrinkage), and the following wafers are incrementally numbered towards the last, “N”, near the tip of the ingot (conical end). The ingots were cut in wafers according to the [111] plane, which was generally not perpendicular to the growth axis of the ingot.

**Figure 6:**
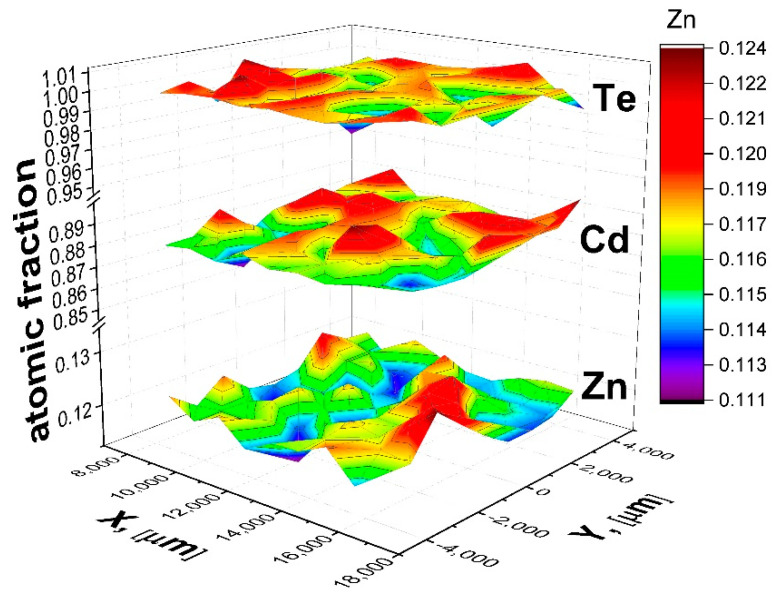
Plot of Cd, Zn, and Te (EPMA) on the 9th wafer surface of CZTL10 ingot.

The atomic fraction (at.) of Cd, Zn, and Te (EPMA) of a [111] CZT wafer is plotted in Figure 6 in which the radial variation of composition is observed. Each ingot nomenclature, as it is shown in Table 1, are the following: CT:CdTe; CZT:CdZnTe; L: 1.66 mm/h; F: 3.22 mm/h; 10:0.10 at. Zn; and 4:0.04 at. Zn.

As an example, the atomic fraction (at.) of Cd, Zn, and Te obtained from EPMA measurements on the surface of wafer 9 of a CZTL10 [111] ingot is plotted in Figure 6. A homogeneous Cd, Zn, and Te distribution is observed, with low deviation from the average atomic fraction, observed as summits and valleys in the two-dimensional distribution. From similar measurements, we obtained the average and dispersion values of the Cd, Zn, and Te composition in each wafer.

#### 3.2.2. Boxplot Diagram Analysis

**Figure 7:**
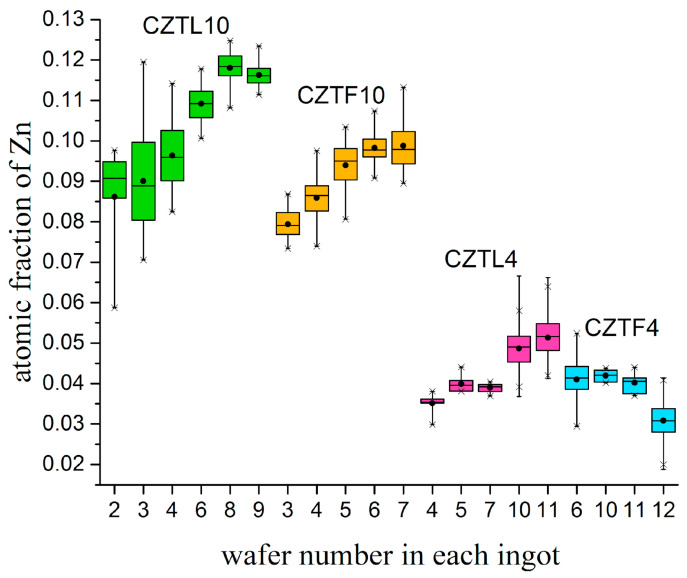
Zn atomic fraction in alloyed CdTe wafers. The total number “n” measurements (population) were on average around 80 for each wafer. This plot was built using Origin (Plot/Statistics/Box Chart).

**Figure 8:**
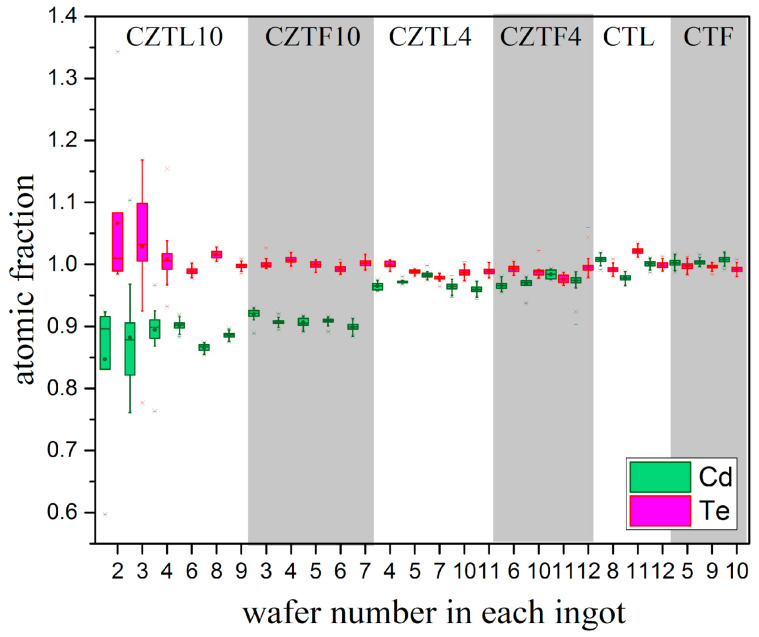
Cd and Te atomic fraction in wafers of all grown ingots. The total number “n” measurements (population) were on average around 80 for each wafer.

The Cd, Zn, and Te atomic fractions are box-plotted in Figure 7 and Figure 8 for several wafers of each grown ingot [34]. Atomic fractions are employed in the plots for a better visualization. Figure 7 shows that the Zn atomic fraction increases from the upper edge towards the ingot tips, except in ingot CZTF4, where the trend is the opposite. Zn is a Cd substitutional dopant, but its much lower mass allows it to be incorporated in the growth of single crystals with a Zn distribution coefficient higher than one, which leads to its segregation during growth and to an inhomogeneous atomic composition in the ingot [9].

As the first three wafers of the CZTL10 ingot (wafer number “2” and “3” in Figure 7 and Figure 8) are very close to the top of the ingot (shrinkage), their values present a lot of dispersion, possibly due to contamination. Ignoring these wafers, the obtained compositions in terms of Cd and Te, are approximately similar to CZTF10 (Figure 8). In terms of Zn (ingots CZTL10 and CZTL4, Figure 7), the lower ingot growth rate gives to this element the possibility to diffuse more, resulting in an increase of Zn concentration in the ingots. It is observed that CZTL4 and CZTF4 ingots are more homogeneous in terms of the three-element compound (Figure 7 and Figure 8) due to a lower Zn concentration [35]. Figure 8 shows that Te concentration in general is closer to 1 in all ingots and Cd atoms are replaced by Zn with some exceptions: Wafer 7 of CZTL4 and wafer 11 of CZTF4.

No significant composition variations in pure CdTe ingots for different growth rates are observed. Moreover, their boxes indicate little dispersion (Figure 8). In some pure CdTe ingots (wafers 8 and 12 of the CTL ingot and the three CTF wafers), the Te atomic fraction is lower than the Cd atomic fraction, indicating the presence of Te precipitates [29].

### 3.3. Detection of Te Precipitates/Inclusions

**Figure 9:**
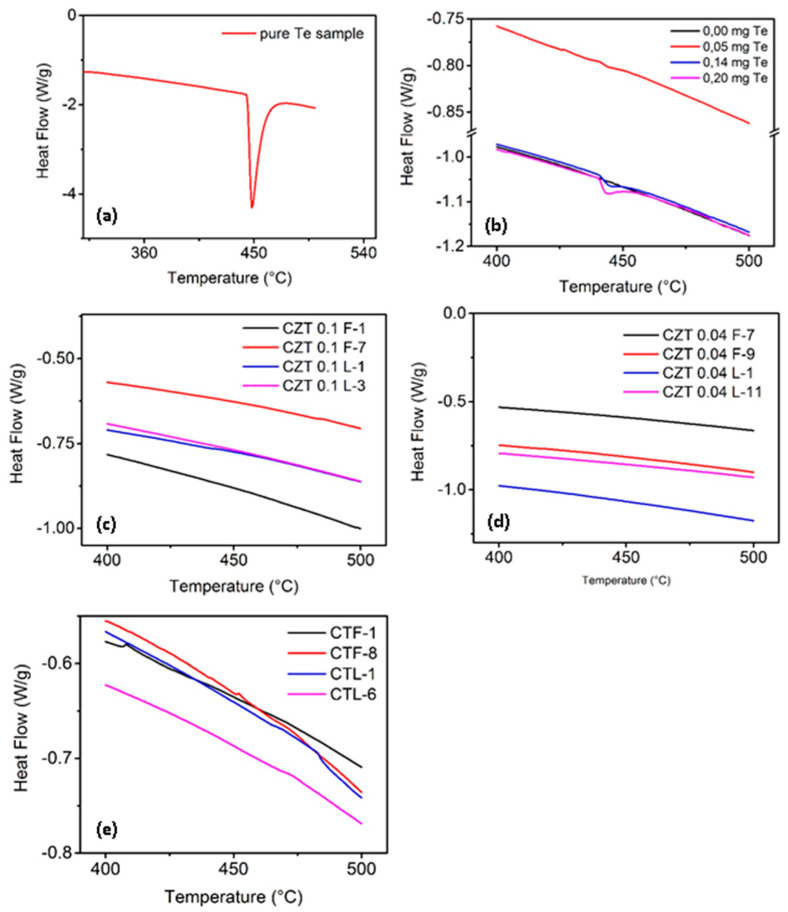
DSC spectra of: (**a**) Pure (s) Te sample; (**b**) Commercial CZT (x = 0.04) with pure (s) Te addition; (**c**) CZT samples doped with 10 at% Zn F (1, 7) and L (1, 3); (**d**) CZT samples doped with 4 at% Zn F (7, 9) and L (1, 11) and (**e**) CdTe grown at 1.66 mm/h (L-1, L-6) and 3.32 mm/h (F-1, F-8).

It was also important to determine the concentration of Te precipitates/inclusions in the grown ingots. For that purpose, DSC tests were performed on samples of all ingots grown in our laboratory at UNIDEF [15,36]. A series of experiments were carried forward with decreasing amounts of Te added to samples of commercial CdZnTe (20 mg of mass in all cases) to determine the sensitivity of the thermal analysis equipment to the presence of Te precipitates/inclusions. Figure 9a shows the DSC result for pure Te, where the peak corresponds to the fusion of that simple element. Figure 9b shows the results of the application of the same technique for commercial CZT, taken as a standard, with different amounts of Te addition. The area where the peak appears due to the fusion of Te is enlarged in the top right box of the figures. The results of the DSC tests for CZT and CdTe samples of ingots grown at UNIDEF are shown in Figure 9c–e. The presence of Te can be perceived in commercial materials when the Te addition is higher than 0.05 mg. The corresponding signal is observed with 0.20 mg, 0.14 mg, and 0.05 mg of Te addition, and is barely perceptible in the last case. It can be concluded that the minimum amount of Te precipitates that can be detected with the conditions under which the tests were carried out is of the order of 0.25% wt/wt.

In no case, the Te fusion peak ostensibly appears. It can, therefore, be stated that the concentration of Te precipitates/inclusions in all these samples is below the limit of 0.25% wt/wt, as mentioned above.

### 3.4. Optical Transmission Analysis

The Te precipitates/inclusions reduce infrared IR transmittance, degrade lattice perfection and increase defect densities in CdZnTe material [37]. Te inclusions significantly influence detectors properties [38,39]. Strains are introduced in the crystalline structure producing lattice distortions that decrease the crystalline optical transmission. This fact also degrades electrical properties [40,41].

**Figure 10:**
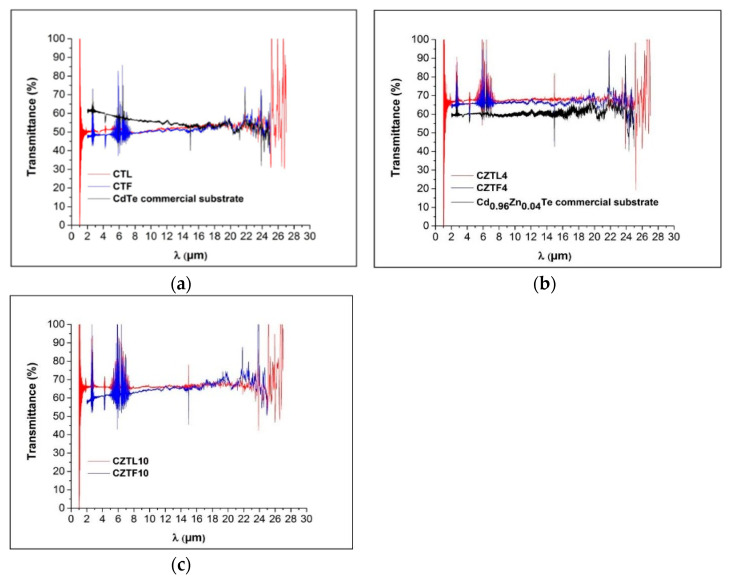
Optical transmission of: (**a**) CdTe; (**b**) Cd_0.96_Zn_0.04_Te; and (**c**) Cd_0.90_Zn_0.10_Te.

Optical transmittance measurements can then be used to determine the amount of defects. We investigated pure CdTe and Cd_0.96_Zn_0.04_Te, grown by Bridgman’s method, and compared the results with those obtained from commercial substrates of the same composition (Figure 10a,b). Meanwhile, Figure 10c only shows the optical transmittance of Cd_0.9_Zn_0.1_Te grown at different rates.

The optical transmission in the pure CdTe (Figure 10a) is only in the range between 47.5 and 55%, meanwhile the case of CdTe alloyed with Zn gives an excellent result since the variation range is between 60 and 70% in a wavelength ranging from 2 to 27 micrometers (Figure 10b,c). It is observed that the decrease of growth rate led to an increase in transmittance, indicating an improvement of grain structure and crystalline quality of the material.

W.J. KIM et al. [42], similar to us, grew the materials by the vertical Bridgman’s method and observed that the IR transmittance gradually decreases from 55% to 5% with a decreasing wave number, attributed to Cd vacancies. This value was improved by annealing in Cd atmosphere, to annihilate Cd vacancies or combine with Te precipitates to form CdTe, reaching 65% of transmittance in all the wave number range. In our case, the quality of the crystals obtained provides transmittance values between 47.5% and 55% in the same wavelength range. The crystal grown at a slower speed has a 2% higher transmittance than the one grown at a higher speed. Therefore, the technique used in our work, although it does not provide the best transmittance values, gives them with less variability. CdTe grown by the vertical gradient freeze method, which is more sophisticated and controlled, reaches values higher than 60% transmittance with little dispersion in the same wavelength range [43].

Our IR Transmission values of CdTe doped with 4 at% Zn can be compared with those of X. Zhang et al. [44], who used the modified vertical Bridgman’s method (MVBM) as the growth technique and obtained transmittance values between 59 and 45% for a similar wavelength range. The authors attributed the low transmittance values to large size Te precipitates and Cd precipitates, but after annealing in a saturated Cd atmosphere, the values rise to 60%. Our results agree with those obtained by J. Zhu et al. [37], who grew the samples by the conventional vertical Bridgman’s technique in a multizone furnace. DSC measurements indicate that a concentration of Te precipitates larger than 0.6 wt% reduces the IR transmittance to values lower than 55%. A concentration lower than 0.35 wt% did not influence the IR transmittance. In our DSC results, no precipitated Te concentrations higher than 0.25% wt/wt were detected, being consistent with our transmittance values, higher than 60%.

CdTe doped with 10 at% Zn (Figure 10c) presents IR transmission values similar or slightly higher than samples grown by vertical Bridgman’s method with three-stage variable growth rates, by Liang et al. [45]. Those ingots, with a slightly convex or concave interface, showed IR transmittance lower than 60%, attributed for the authors to Te precipitates. Moreover, our IR measurements are higher than the 61% obtained by F. Yang et al. [26] in Cd_0.9_Zn_0.1_Te ingots with 10 ppm (at.) Indium dopant grown by the pressure-controlled Bridgman’s method. They also attributed these results to Te precipitates.

Overheating influences the nucleation and growth phenomena of the semiconductor material since it breaks Te aggregates and the concentration of this element is reduced by preventing Cd vacancies formation and condensation [29]. It was proposed that in order to grow pure CdTe, an overheating 100 K above the melting point is necessary, greater than for the CdTe alloyed with Zn [46]. In our case for both, pure CdTe and CdTe alloyed with Zn, the same overheating was employed (approximately 20 °C above the melting point of each of the substances). This could be a reason for the differences that appear in our transmission spectra of pure CdTe compared to commercial samples. In the growth of the CdTe alloyed with Zn, the overheating of 20 °C above the melting point produced in our case a good result, since the transmittance values were above 60%.

The Zn addition in the CdTe matrix is important to improve its lattice structure, since a material hardening effect is achieved. For example, the values of the average theoretical critical shear stress (CRSS), obtained through microhardness tests, are 6.4 MPa and 27.4 MPa respectively for CdTe and Cd_0.96_Zn_0.04_Te [12,47].

Results show that CdTe alloyed with Zn has a higher optical transmission than pure CdTe, which could indicate that the addition of Zn reduces the amount of defects. The crystalline quality of pure CdTe could be improved by annealing the treatment after growth [48], reducing the amount of defects.

### 3.5. CZT Energy Band (Eg) Measurement

**Figure 11:**
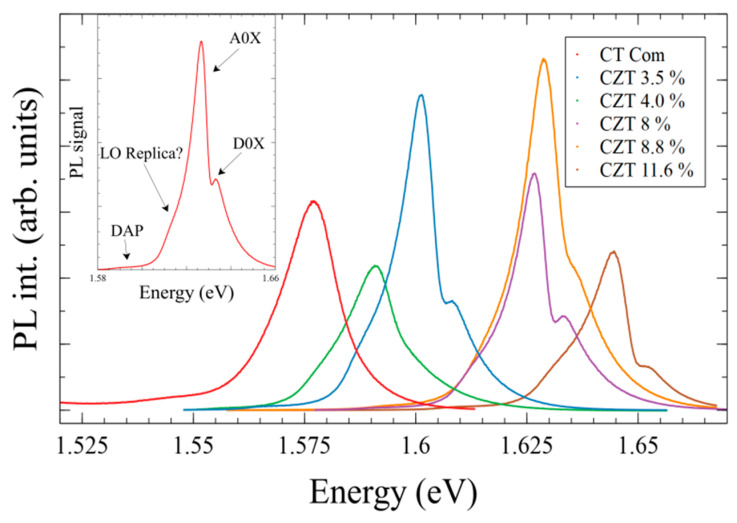
Photoluminescence spectra of wafers with different Zn composition, obtained at 76 K. Inset: Peak assignment in the spectra of the wafer with 8.8 at% Zn.

Experimental values of band gap energy (Eg) of CZT wafers were obtained by photoluminescence (PL) technique at liquid Nitrogen temperatures (76 K with 5 K temperature accuracy). In a typical PL spectra of CZT at low temperature, the donor-bound exciton (D°,X hereafter D0X) and acceptor bound exciton (A°,X hereafter A0X) transitions, as well as phonon replica, among others, can be observed [49]. The inset of Figure 11 shows a PL spectrum of CZT with 0.088 at. Zn composition obtained at 76 K. Although at this temperature not all peaks are well distinguished, the (A0X) and (D0X) are clearly observed. Some other peaks at lower energies—probably phonon replica—can be inferred. The main part of Figure 11 shows the spectra obtained from most of the measured wafers.

The value of the gap can be obtained from the transition energy assuming that the strongest peak corresponds to the transition associated with the acceptor (A°,X) and that the peak is 16 meV below the gap [50]. The composition value of each of the wafers was determined by the average of EPMA results, as explained in Section 3.2.1. EPMA results show that the location of the wafer in the ingot affects its composition. This is explained by a certain degree of inhomogeneity in the ingot as a result of the cooling process that causes a composition gradient along the ingot [29]. The wafer, on the opposite, is practically homogeneous in its composition.

**Figure 12:**
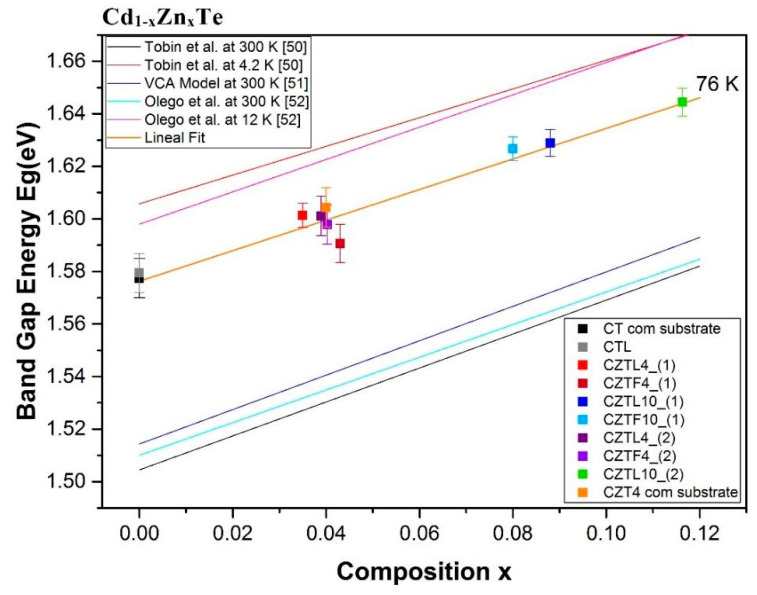
Eg as a function of the Zn atomic fraction in the concentration range of 0 ≤ x ≤ 0.10. The points, obtained in this work, are compared with some models from the literature (lines).

Eg values are strongly dependent on the composition, as shown in Figure 12, so that the EPMA results can be corroborated by PL measurements. To check the homogeneity of the wafers, Eg was obtained from the average of about 10 different positions on the wafer surface, observing a variation of less than 1%. It can be concluded that the samples are radially homogeneous. On the contrary, comparing wafers of the same ingot, it is observed that, as the Zn composition increases along the ingot axis, the energy value of the band gap also increases.

Energy gap values Eg for different Zn compositions are plotted in Figure 12, together with a compilation of results found in the literature [50,51,52]. Each of the dots in the graph corresponds to a wafer and the error is estimated from the peak fitting. As clearly observed, the experimental Eg values, measured at 76 K, follow a linear regression comparable to those proposed in the literature, where the temperature is responsible for the stiff shift. Our data are then consistent with a temperature of 76 K.

## 4. Conclusions

It was experimentally found that the chemical etching solution (Nakagawa’s solutions) could be used for dislocations observation of the [111] plane in all compounds, from pure CdTe and also for Cd_0.96_Zn_0.04_Te and Cd_0.9_Zn_0.10_Te.

In all ingots grown by Bridgman’s method, the average values of dislocation density and misorientation between adjacent subgrains are comparable.

Linear defects increase in wafers approaching the upper and lower ends of the ingots, regardless of chemical concentration. These wafers must be discarded. In all cases, during growth, impurity concentrations increased in these regions due to the temperature gradients that occur during cooling.

Ingots grown at 3.32 mm/h have four or five grains of different crystallographic orientations. Meanwhile, ingots grown at 1.66 mm/h have a maximum of three grains, indicating a higher crystal quality. In both cases, ingots present greater composition homogeneity in the central zone.

Box and extension graphs obtained with EPMA measurement results reveal both axially and radially concentration gradients in the CZT wafers. In ingots of CdTe alloyed with Zn, the atomic fraction of Zn increased from the upper to the lower end of ingots and the inhomogeneity degree increased with increasing Zn concentration. In CZTL10 and CZTF10 ingots, the composition of Cd and Te was independent of the used growth rates. In CZTL4 and CZTF4 ingots, it was observed that at a lower speed growth the three elements were more homogeneous in composition. Cd and Te concentrations were very similar in the CTL and CTF ingots, although in both cases Te decrease was in general observed, indicating the precipitates formation of this element.

In all samples, the concentration of Te precipitates/inclusions was below the detectivity limit of the DSC measuring equipment (0.25% wt/wt). It was also observed that this concentration was lower than the minimum level from which the infrared transmittance of CZT decreased, which corresponded to 0.6% wt/wt. The DSC technique did not detect Te precipitates/inclusions.

CdTe wafers from ingots alloyed with 4 at% and 10 at% Zn grown at 1.66 mm/h, had the best-infrared transmission spectra with an average value of 67%, higher than commercial samples. Ingots grown at a higher speed of 3.32 mm/h also exhibited higher transmittance than commercial samples but had lower crystalline quality. Pure CdTe grown at both speeds had a transmittance below 55%. This decrease is attributed to the higher concentration of defects. The addition of Zn in the CdTe matrix improved its structure and determined the highest observed optical transmission.

The band gap energy obtained by photoluminescence (PL) experiments increased when increasing the Zn content in the CdTe matrix. Eg presented little variation with respect to the growth rate change of ingots of the same composition, although, for most of the wafers, Eg was superior in the ingots grown at a lower speed. Eg was practically constant at different positions of the wafer and its variation along the ingot axis was very low. In addition, the obtained Eg energies were consistent with the composition values obtained by EPMA.

These properties support the use of these materials to manufacture electronic devices of comparable sensitivity. In addition, costs are reduced since each one of the ingots can contribute with a greater volume of the material, except the ingots extremes due to the presence of impurities and lack of crystallinity.

We demonstrated that the Bridgman’s method can be used to grow CZT suitable for detectors with high sensitivity with the technological advantage of increasing the volume of the high quality grown material and the consequent reduction in fabrication costs.

## Figures and Tables

**Table 1:** CZT atomic fraction, growth rate, grains number, average dislocations density (δ), and average contiguous subgrains misorientation (ϕ) are shown for each ingot. The average dislocations density (δ) and the average contiguous subgrains misorientation (ϕ) are also shown for some [111] wafers.

Ingot Number	Zinc Content in Cd_1−x_Zn_x_Te (0 ≤ x ≤ 0.1)	Growth Rate (mm/h)	Number of Grains	Wafer Number	Wafer Average Density δ (cm^−2^) (Nakagawa)	Ingot Average Density δ (cm^−2^) (Nakagawa)	Wafer Average ϕ(″)	Ingot Average ϕ(″)
1	CdTe CTL x = 0	1.66	2	4	4.5 × 10^6^	2.6 × 10^6^	32	30
				7	2.3 × 10^6^		32	
				8	1.9 × 10^6^		26	
				11	2.9 × 10^6^		37	
				12	1.6 × 10^6^		23	
2	CdTe CTF x = 0	3.32	4	4	1.4 × 10^6^	1.9 × 10^6^	23	32
				5	1.5 × 10^6^		30	
				6	1.6 × 10^6^		30	
				9	3.1 × 10^6^		44	
3	Cd_0.96_Zn_0.04_Te CZTL4 x = 0.04	1.66	2	4	4.7 × 10^6^	3.2 × 10^6^	35	29
				5	1.9 × 10^6^		35	
				7	2.0 × 10^6^		17	
4	Cd_0.96_Zn_0.04_Te CZTF4 x = 0.04	3.32	5	10	1.0 × 10^6^	8.0 × 10^5^	27	24
				11	6.0 × 10^5^		21	
5	Cd_0.9_Zn_0.1_Te (CZTL10) x = 0.10	1.66	2	1	8.0 × 10^5^	1.2 × 10^6^	20	23
				2	7.6 × 10^5^		22	
				3	7.0 × 10^5^		16	
				4	7.0 × 10^5^		18	
				6	1.4 × 10^6^		36	
				8	2.2 × 10^6^		26	
6	Cd_0.9_Zn_0.1_Te (CZTF10) x = 0.10	3.32	4	3	9.7 × 10^5^	7.7 × 10^5^	24	23
				4	7.9 × 10^5^		35	
				5	4.3 × 10^5^		25	
				6	1.1 × 10^6^		36	
				7	4.9 × 10^4^		5	
				11	5.2 × 10^5^		13	

## Data Availability

The data presented in this study are available on request from the corresponding author.
